# Treating addiction with psychedelics - are we waking up?

**DOI:** 10.1192/j.eurpsy.2021.1536

**Published:** 2021-08-13

**Authors:** J. Miranda, M. Barbosa, I. Figueiredo, P. Mota, A. Tarelho

**Affiliations:** 1 Mental Health Department, Centro Hospitalar de Leiria, Leiria, Portugal; 2 Mental Health Department, Hospital Professor Doutor Fernando Fonseca, Lisboa (Amadora), Portugal; 3 Departamento De Psiquiatria E Saúde Mental, Centro Hospitalar do Tâmega e Sousa, Guilhufe, Portugal

**Keywords:** psychedelics, Addiction, Substance Use Disorder

## Abstract

**Introduction:**

Classic psychedelics have been administered in sacramental contexts since ancient times. They were of prominent interest within psychiatry and neuroscience in the 1950s to 1960s, but the association between classic psychedelics and the emerging counterculture put an end to their research. Modern research with classic psychedelics has reinitiated interest in the treatment of both cancer-related distress and addiction, with really promising results.

**Objectives:**

We aim to provide a review about history and new insights regarding research with psychedelics specially as treatment of addictive disorders.

**Methods:**

A framing analysis of articles, searched on Pubmed (articles between 2010-2020) with the key words: “ psychedelics”, “psilocybin”, “substance use disorder”, “addiction”.

**Results:**

Classic psychedelics are 5HT2AR agonists such as LSD, mescaline, and psilocybin. They were shown to occasion mystical experiences, which are experiences reported throughout different cultures and religions involving a strong sense of unity. These experiences are scientifically important because they appear to cause abrupt and sustained changes in behavior and perception, that can be very useful in the substance use disorder field. From this analysis is possible to understand that the use of psychadelics in the treatment of some addictions is currently at an early stage of research. However, they show interesting results with no clinically significant adverse events when risk individuals are excluded.

**Conclusions:**

In comparison to psychedelic research about cancer-related psychological distress, studies with addictions are less developed, but if they continue to suggest safety and efficacy, may be the use of psilocybin for the treatment of specific addiction can happen in a close future.

